# Ophthalmology

**Published:** 1898-04

**Authors:** Alvin A. Hubbell

**Affiliations:** Buffalo, N. Y., Professor of ophthalmology in Niagara University Medical College


### OPHTHALMOLOGY.

CONDUCTED BY ALVIN A. HUBBELL, M. D., Buffalo, N. Y.,
Professor of ophthalmology in Niagara University Medical College.

### PAINLESS EYE OPERATIONS.

BATES, W. H., of New York, (*New York Med. Jour.*, October 16, 1897,) calls attention to the value of a solution of the extract of the suprarenal capsule as a supplement to cocaine. He says that the latter does not prevent pain in many tenotomies and advancements, and in most lachrymal operations, and that inflamed eyes resist the action of cocaine. During the past three years he has operated in more than 100 cases in which the extract was as necessary as cocaine in securing complete anesthesia. He has operated upon inflammatory glaucoma painlessly, using the suprarenal extract to relieve the congestion, so that cocaine was able to anesthetise. He states that Darier, of Paris, has since reported painless iridectomies in these cases from the use of the suprarenal extract and cocaine. He claims that the extract is not objectionable in any way, being neither irritating nor poisonous ; it is merely a powerful astringent. The solution must be properly prepared. The solution is prepared by mixing about ten grains of the dried extract powdered gland with half a drachm of water and filtering ; it must be prepared just before the operation, since it soon spoils when exposed to the air at the ordinary temperature. Nothing else should be mixed with it. A few drops are used alternately with the cocaine solution.

The writer also advocates the use of a hot salt water (ʒi to one pint) douche, about 115°, to the operated part of the eye, as soon as the operation is finished, while the eye is still under the influence of cocaine. He uses a fountain syringe and employs about three quarts of water. He claims that this prevents the occurrence of pain and soreness after an operation.

---

### THE PURSE STRING SUTURE OF THE CONJUNCTIVA IN CASES OF SOLUTION OF CONTINUITY OF THE CORNEA.

ROHMER, M., of Paris, (*Annales d' Oculistique*, November, 1897,) cites twenty-three cases of the above nature treated by himself with the purse string suture of the conjunctiva, according to the de Wecker method, and one treated by Meyer's method of conjunctival transplantation, with but one failure. He also adds two cases previously reported by Meyer, one by Weiss, of Heidelberg, one of a burn of the ciliary region, by Norman Hansen, and one of a corneal fistula, seen by Bourgeois; all of which were treated successfully by conjunctival transplantation.

He prefers the method of de Wecker, lays stress on securing the subconjunctival tissue, and insists that this adheres only to the injured area. He has found that the conjunctiva should be, if necessary, dissected back as far as the muscular attachments.

As the indications for the operation he names (1) recent wounds of the cornea, either simple or those which are accompanied with irreducible hernia of the iris, the iris to be first resected; (2) old synechiæ, where the iris is on a level with the surface of the cornea; (3) limited corneal or sclero-corneal staphylomata; (4) corneal ulcers that are complicated with hernia of the iris; (5) corneal fistulæ and irido-cyclitis occurring from infection through a cicatrix; and (6) cases of retarded cicatrisation following cataract operation.—*Annals of Ophthalmology*, January, 1898.

---

### ORIGIN AND NATURE OF THE VITREOUS BODY.

Tornatola, M. S., (*Revue Generale d' Ophthalmologie*, December 31, 1897,) claims that the vitreous body is composed in part of fine fibrils (either separate or joined into bundles), of large nucleated mesodermic cells which form and nourish the hyaloid artery, and which disappear with the artery; and in part of smaller bodies, which are either migratory cells or old débris of the larger cells. The fibers can be traced directly to the protoplasm of certain retinal cells, from whence they spring.

The vitreus, he believes, is, therefore, in accordance with his work, of ectodermic origin and with the exception of the cells, which are not necessarily parts of the organ itself, not mesodermic, as is generally supposed.—*Annals of Ophthalmology*, January, 1898.

